# The economically important thrips from Malaysia, with a key to species (Thysanoptera, Thripinae)

**DOI:** 10.3897/zookeys.810.28457

**Published:** 2018-12-20

**Authors:** Yong Foo Ng, J Saiful Zaimi

**Affiliations:** 1 Centre for Insect Systematics (CIS), Universiti Kebangsaan Malaysia, 43600 Bangi, Selangor, Malaysia Universiti Kebangsaan Malaysia Bangi Malaysia; 2 Malaysian Agricultural Research and Development Institute, MARDI Cameron Highlands 39007 Tanah Rata, Pahang, Malaysia Malaysian Agricultural Research and Development Institute Tanah Rata Malaysia

**Keywords:** Economically important, Malaysia, Thripinae, thrips, Thysanoptera

## Abstract

An illustrated key is provided to the economically important Thripinae (Thysanoptera) of Malaysia, together with a checklist and information on hosts and distributions. Information about the diversity and pest status for these Thripinae is provided, together with the prominent character states that are useful for recognising each species.

## Introduction

Of the 6200 thrips species worldwide (ThripsWiki 2018), most of the economically important pest species are members of the subfamily Thripinae. Notorious for their broad range of hosts, members of this subfamily are small, pale to dark brown in colour, and often occur in large populations. The common injuries are fruit scarring and flower decolouration, which directly lowers the aesthetic value and market price of crops. Moreover, crops infested by thrips may be banned from importation into countries with strict biological quarantine procedures, such as USA, Australia, Japan, and European countries. Other indirect damages include the reduction of crop yields due to foliage and flower dropping caused by the insect’s feeding activities. Several species of Thripinae are also the only known vectors of important plant tospoviruses (Moritz et al. 2003), such as the Tomato Spotted Wilt Virus (TSWV), Impatiens Necrotic Spot Virus (INSV), and other tospoviruses as reported by Rotenberg et al. (2015).

Members of Thripinae can be identified only with certainty after careful mounting onto microscope slides. Partly due to a lack of trained workers and quick identification keys, the pest management practices for these minute insects in Malaysian agriculture and horticulture industry have been impeded, compared to temperate countries where thrips are generally better studied. For instance, although commonly occurring in a wide range of ornamental flowers and vegetable crops, there has been no identification key to the range of species found on crops in Malaysia. This paper introduces the economically important pest species of the subfamily Thripinae recorded from Malaysia, together with an illustrated key to these common pests. A list of host plants and distributions for each species are also included, and information on morphology and biology is also explained wherever possible. A key to the 65 genera of Thripinae recorded from South East Asia was provided by Mound and Ng (2009), and a key to the 23 species of the genus *Thrips* recorded from Peninsular Malaysia was provided by Mound and Azidah (2009).

## Material and methods

The specimens studied were obtained by field sampling in Malaysia. Methods used for collecting were by hand-searching and beating. Samples collected were processed onto permanent microscopic slides in Canada Balsam, after maceration in 5% NaOH and dehydration through a series of alcohols of 70%, 80%, 90% and absolute. These slides were then dried at 45 °C on a slide warmer, before being transferred into an oven for at least one month. All studied slides are deposited in the Centre for Insects Systematics (**CISUKM**), Universiti Kebangsaan Malaysia. Identifications and diagnoses were carried out with a differential interference contrast (DIC) microscope (Olympus BX41) as indicated in the photomicrographs that accompany the key provided here.

## Results

### Key to the economically important thrips from malaysia

**Table:** 

1	Abdominal tergites with numerous rows of microtrichia (at least on the lateral third) (Fig. 10); antennae with 8 segments; body length usually about 1mm or shorter	*** Scirtothrips dorsalis ***
–	Abdominal tergites without numerous rows of microtrichia (Figs 8, 9); antennae with 7 or 8 segments (Fig. 7); body length usually more than 1 mm	**2**
2	Abdominal tergites VI–VIII with a pair of ctenidia laterally (Figs 8, 9)	**3**
–	Abdominal tergites VI–VII without a pair of ctenidia laterally	**10**
3	Ocellar setae pair I present (Figs 1, 4, 5); Pronotal anterior margin with 1 or 2 pairs of long setae	**4**
–	Ocellar setae pair I absent (Figs 2, 3); Pronotal anterior margin without any pairs of long setae	**6**
4	Tergite VIII of female without a posteromarginal comb of microtrichia; head with ocellar setae pair III close together and arising within ocellar triangle (Fig. 5)	*** Franklliniella schultzei ***
–	Tergite VIII of female with posteromarginal comb of microtrichia; ocellar setae pair III arising just outside anterior margins of ocellar triangle (Fig. 4)	**5**
5	Metanotum with a pair of campaniform sensilla (Fig. 17); postocular setae pair IV about as long as distance between hind ocelli	*** Frankliniella occidentalis ***
–	Metanotum without a pair of campaniform sensilla; postocular setae IV short, not longer than distance between hind ocelli (Fig. 4)	*** Frankliniella intonsa ***
6	Abdominal tergites with triangular lobes of craspedum on posterior margin (Fig. 8); prosternal basantra with several setae (Fig. 13)	*** Microcephalothrips abdominalis ***
–	Abdominal tergites without craspedum (cf. Fig. 9); prosternal basantra never with setae	**7**
7	Ocellar setae II longer than setae III; postocular setae setae I and III usually long	*** Stenchaetothrips biformis ***
–	Ocellar setae II longer than setae III; postocular setae setae I and III usually short	**8**
8	Metanotal median setae arising far the behind anterior margin (Fig. 16); abdominal sternites IV–VI without discal setae (Fig. 12)	*** Thrips palmi ***
–	Metanotal median setae arising at anterior margin (cf. Fig. 17); abdominal sternites IV–VI with discal setae (Fig. 11)	**9**
9	Postocular setae II stout, more than 0.5 as long as the distance between the bases of setae I and II (Fig. 2); forewing clavus apical setae usually as long as or longer than subapical (Fig. 19)	*** Thrips hawaiiensis ***
–	Postocular setae II small, less than 0.5 as long as the distance between the bases of setae I and II (Fig. 3); forewing clavus apical setae usually shorter than subapical (Fig. 18)	*** Thrips florum ***
10	Ocellar setae I absent; abdominal tergite VIII posterior margin with microtrichial comb present medially (Fig. 24)	**11**
–	Ocellar setae I present; abdominal tergite VIII posterior margin with or without microtrichial comb present medially (Fig. 24, 25)	**12**
11	Pronotum without prominent postero-angular setae (Fig. 21); metafurcal spinula weak or absent (Fig. 14)	*** Dichromothrips corbetti ***
–	Pronotum with one pair of long postero-angular setae (Fig. 20); metafurcal spinula well developed (Fig. 15)	*** Dichromothrips smithi ***
12	Abdominal tergite VIII posterior margin with microtrichial comb present medially (cf Fig. 24)	*** Ceratothripoides brunneus ***
–	Abdominal tergite VIII posterior margin without microtrichial comb medially (Fig. 25)	**13**
13	Abdominal sternite VII posteromarginal setae arising in a row along posterior margin (Fig. 23); basal half antennal segments IV and V yellow (Fig. 6)	*** Megalurothips typicus ***
–	Abdominal sternite VII posteromarginal median setae arise in front of posterior margin (Fig. 22); antennal segments IV and V entirely brown (Fig. 7)	*** Megalurothips usitatus ***

**Figures 1–12.**
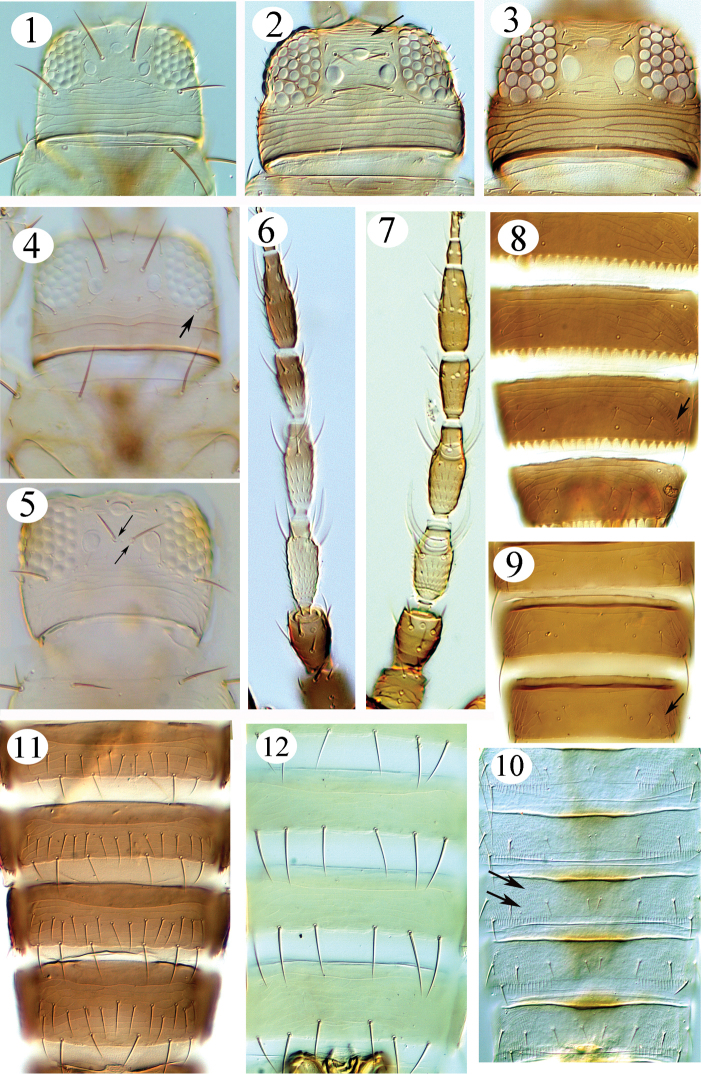
Head: **1***Frankliniellaoccidentalis*, **2***Thripshawaiiensis*, **3***Thripsflorum*, **4***Frankliniellaintonsa*, **5***Frankliniellaschultzei*. Antenna: **6***Megalurothipsusitatus*, **7***Megalurothripstypicus.* Abdominal tergite: **8***Microcephalothripsabdominalis*, **9***Thripshawaiiensis*, **10***Scirtothripsdorsalis*. Abdominal sternite: **11***Thripshawaiiensis*, **12***Thripspalmi*.

**Figures 13–25.**
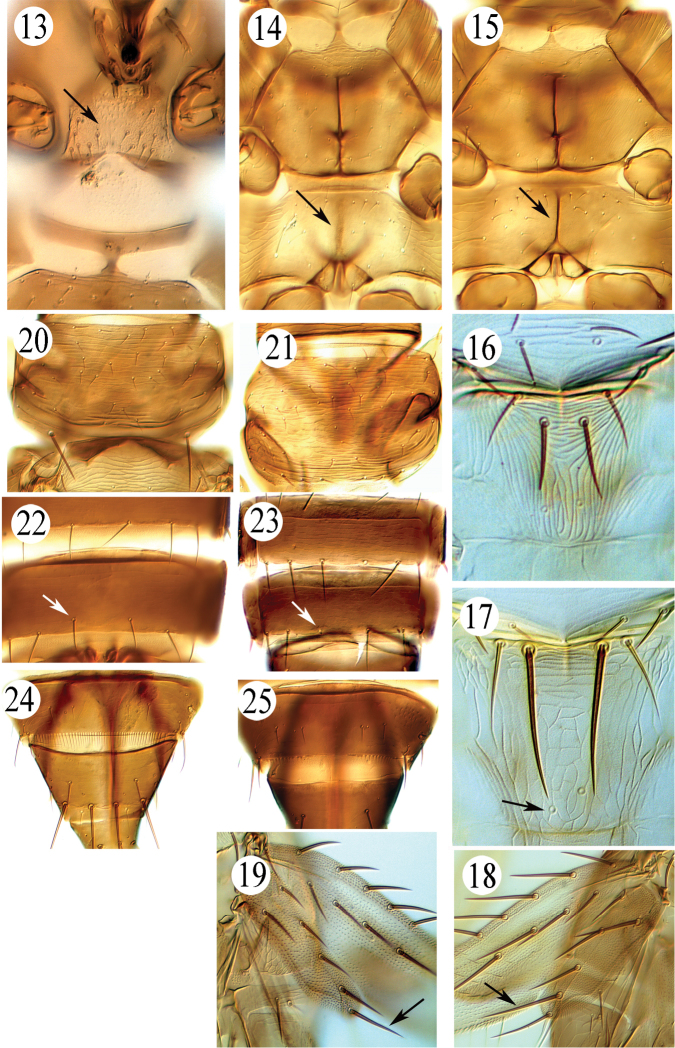
Prosternal basantra: **13***Microcephalothripsabdominalis*. Meso- and Metasternum: **14***Dichromothripscorbetti*, **15***Dichromothripssmithi.* Metanotum: **16***Thripspalmi*, **17***Frankliniellaoccidentalis.* Forewing clavus: **18***Thripsflorum*, **19***Thripshawaiiensis.* Pronotum: **20***Dichromothripssmithi*, **21***Dichromothripscorbetti.* Abdominal sternite VII: **22***Megalurothipsusitatus*, **23***Megalurothipstypicus.* Abdominal tergite VIII: **24***Dichromothripscorbetti*, **25***Megalurothipstypicus*.

### *Ceratothripoidesbrunneus* Bagnall

Five species are recognized in this genus, all from the Old World (Mound and Nickle 2009), but only *C.brunneus* has been found in Malaysia. A closely related species, *C.claratris*, has been reported as a pest (Murai 2000) and vector of tospoviruses on tomatoes in Thailand (Halaweh and Poehling 2009). This species was not detected in our collections, nor was it reported during earlier surveys (Mound and Azidah 2009). The species *C.brunneus* is from Africa, but has been found recently in Peninsular Malaysia on a range of different plant species (Table 1).

**Table 1.** List of economically important thrips, their host plants and distribution in Malaysia.

No.	Thrips species	Host plant		Distribution
		Family	Species	
1.	* Ceratothripoides brunneus *	Acanthaceae	* Strobilanthes crispus *	Pahang, Johor, Penang
		Apocynaceae	* Allamanda oenotheraefolia *	
		Lamiaceae	* Orthosiphon aristatus *	
		Rubiaceae	*Ixora* sp.	
		Verbenaceae	* Stachytarpheta mutabilis *	
2.	* Dichromothrips corbetti *	Orchidaceae	*Aracnis* sp.	Penang, Sarawak
			* Arundina graminifolia *	
			*Cattelya* spp.	
			*Dendrobium* spp.	
			*Phalaenopsis* spp.	
			*Vanda* spp.	
3.	* Dichromothrips smithi *	Orchidaceae	* Arundina graminifolia *	Pahang, Sarawak
4.	Frankliniellaintonsa*	Alliaceae	* Apapanthus campanulatus *	Cameron Highlands, Pahang
		Asteraceae	*Dahlia* sp.	
		Balsaminaceae	Impatiens sp. var. wallerana	
			* Impatiens balsamina *	
		Caryophyllaceae	*Gypsophila* sp.	
		Fabaceae	*Vignasinensis var. sesquipedalis*	
		Iridaceae	*Gladiolus* sp.	
		Orchidaceae	* Arundina graminifolia *	
		Plantaginaceae	*Antirrhiinum* sp.	
		Rosaceae	* Fragraria ananassa *	
			*Rosa sp.*	
		Solanaceae	* Solanum melongena *	
		Verbenaceae	*Verbena* sp.	
5.	Frankliniellaoccidentalis*	Alliaceae	* Agapanthus campanulatus *	Penang, Cameron Highlands
		Asteraceae	* Chrysanthemum morifolium *	
			*Gerbera* sp.	
			*Tagetes* sp.	
			* Tanacetum parthenium *	
		Iridaceae	*Gladiolus* sp.	
		Lamiaceae	* Salvia farinacea *	
		Plantaginaceae	*Antirrhinum* sp.	
		Rosaceae	*Rosa* sp.	
6.	Frankliniellaschultzei*	Euphorbiaceae	* Jatropha integerrima *	Melaka
		Nyctaginaceae	*Bougainvillea* sp.	
		Plumbaginaceae	* Plumbago auriculata *	
7.	* Megalurothrips usitatus *	Fabaceae	* Vigna sinensis *	Perak, Penang, Pahang, Selangor
		Fabaceae	* Erythrina fusca *	
		Malpighiaceae	* Tristellateia australasiae *	
8.	* Megalurothrips typicus *	Fabaceae	* Canavalia rosea *	Perak, Penang
		Moraceae	* Artocarpus champeden *	
		Passifloraceae	* Turnera ulmifolia *	
9.	* Microcephalothrips abdominalis *	Acanthaceae	* Pseuderanthemum carruthersii var. reticulatum *	Johor, Penang, Pahang
		Asteraceae	* Chrysanthemum indicum *	
			*Cosmos* sp.	
			* Cosmos caudatus *	
			* Sphagneticola trilobata *	
	* Microcephalothrips abdominalis *	Asteraceae	* Tagetes patula *	Johor, Penang, Pahang
			* Tridax procumbens *	
		Heliconiaceae	*Heliconia* sp.	
		Orchidaceae	*Oncidium* sp.	
		Verbenaceae	* Clerodendrum paniculatum *	
10.	Scirtothripsdorsalis*	Anacardiaceae	*Mangifera* sp.	Penang, Perak, Kedah, Terengganu, Melaka, Selangor
		Fabaceae	* Caesalpinia pulcherrima *	
		Fabaceae	* Cassia fistula *	
			* Pithecellobium jiringa *	
		Fabaceae	* Peliophorum pterocarpum *	
		Myrtaceae	* Psidium guajava *	
		Rosaceae	* Rosa alba *	
		Solanaceae	* Capsicum annuum *	
			* Solanum torvum *	
11.	* Thrips hawaiiensis *	Anacardiaceae	*Mangifera* sp.	Selangor, Pahang, Terengganu, Melaka
		Amaryllidaceae	* Hymenocallis speciosa *	
		Araceae	* Zantedeschia aethiopica *	
		Arecaceae	* Areca catechu *	
			*Dahlia* sp.	
		Asteraceae	* Aster dumosus *	
		Euphorbiaceae	* Jatropha integerrima *	
		Leguminosae	* Caesalpinia pulcherrima *	
		Myrtaceae	* Psidium guajava *	
		Nyctaginaceae	*Bougainvillea* sp.	
		Orchidaceae	* Arundina graminifolia *	
		Plantaginaceae	*Antirrhunum* sp.	
		Rosaceae	* Fragraria ananassa *	
			* Prunus perisica *	
			*Rosa* sp.	
		Verbenaceae	* Clerodendrum paniculatum *	
12.	* Thrips florum *	Apocynaceae	* Plumeria rubra *	Kedah, Penang
		Verbenaceae	* Clerodendrum paniculatum *	
			* Lantana camara *	
13.	Thripspalmi*	Apocynaceae	* Allamanda oenotheraefolia *	Selangor, Pahang, Melaka
			* Plumeria rubra *	
		Asteraceae	*Chrysanthenum* sp.	
			* Cosmos sulphurous *	
			* Sphagneticola trilobata *	
			*Tagetes* sp.	
			* Tagetes patula *	
		Lamiaceae	* Salvia farinaceae *	
		Nyctaginaceae	*Bougainvillea* sp.	
		Orchidaceae	*Aracnis* sp.	
			* Arundina graminifolia *	
			*Dendrobium* spp.	
			*Vanda* spp.	
		Plumbaginaceae	* Plumbago auriculata *	
		Solanaceae	* Solanum torvum *	

*- Vector of Tospovirus (Mound 1996; Mound 2002; Rotenberg et al. 2015).

**Recognition**: *Ceratothripoidesbrunneus* is a large brown species; body largely brown except fore tibiae and antennal segment III paler brown, forewing uniformly brown; abdominal segment VIII posterior margin with complete comb of microtrichia; mesonotum and metanotum without pair of campaniform sensilla; male abdominal sternites III–VII with many small pore plates; male abdominal tergite IX without drepanae. This species is similar in structure to the species of *Megalurothrips*, *Pezothrips*, *Odontothrips*, and *Odonthripiella*, and these share many characteristics including a pair of dorso-apical setae on the first antennal segment. In this paper, this species and the legume-flower thrips (*Megalurothrips*) are distinguished based on the microtrichial comb on abdominal tergite VIII. Recognition of *Ceratothripoides* is easy if males are available, because the sternites have numerous pore plates (Mound and Nickle 2009).

### *Dichromothripscorbetti* (Priesner)

Species of the genus *Dichromothrips* are usually found on orchids. In Peninsular Malaysia, only two species have been recorded so far, although three further species are recorded from western Malaysia - *D.borneensis*, *D.nigricornis* and *D.major*. The species *D.corbetti* originated from the Oriental Region but is now introduced worldwide via cultivated orchids. It was described from *Vandajoaquim* in Kuala Lumpur (Mound 1976), but is known from a wide range of Orchidaceae including *Dendrobium* spp., *Arundina* spp., *Cattelya* spp., and *Vanda* spp. from various localities in lowland Malaysia (Table 1).

**Recognition**: Large and dark brown species, forewing base paler, tarsi and tibial apex yellow; antennal segments brown, segment III and IV with apices elongate. Pronotum without long posteroangular setae, and metafurcal spinula diffuse or weakly defined. These characters are useful in distinguishing the species from the other members of the genus.

### *Dichromothripssmithi* (Zimmermann)

The original material of this species is apparently lost (Mound 1976), and the species is recognized based on female specimens from Taiwan identified by H. Priesner. Recently, it has been reported as an invasive species in US on bamboo orchids (*Arundina* spp.) from Malaysia, Thailand and Taiwan (Department of Agriculture and Plant Industry Division 2010), and as a pest of orchids in Korea (Lee et al. 2002). There are specimens in the CSIRO collection, Canberra, Australia labelled as being associated with damage to Vanilla plants in India. In Malaysia, this species has been taken only from *Arundinagraminifolia* (Bamboo orchid) from Pahang and Sarawak with its distribution restricted to highlands.

**Recognition**: This large, dark brown species is similar in body colour to *D.corbetti*. It is distinguished by the fore wing with the subapical area paler, the pronotum with long posteroangular setae, and the metafurcal spinula well developed.

### *Frankliniellaintonsa* Trybom

This species is known as the European Flower Thrips, due to its wide distribution in Europe, but it causes less damage to horticulture than *F.occidentalis*. Both species share many morphological characteristics, but *F.intonsa* can be recognized by the shorter postocular setae, and the absence of paired campaniform sensilla on the metanotum. Both species also seem to share similar habitats, such that they have been recorded in Peninsular Malaysia only from highlands, but from a wide range of flowers in various plant families, including Plantaginaceae, Alliaceae, Verbenaceae, Orchidaceae, and Asteraceae.

**Recognition**: Body colour brown, tibiae and tarsi yellow; antennal segments III and IV yellow; forewing pale with dark setae. All species of *Frankliniella* have the forewing first and second veins with complete setal rows, the head with ocellar setae pair I present, and the pronotum with four pairs of long major setae. Tergite VIII posterior margin with comb of microtrichia in females but not in males.

### *Frankliniellaoccidentalis* (Pergande)

This species is known as the Western Flower Thrips, and although originally from the South Western states of the USA it is now found worldwide (Moritz et al. 2001). *Frankliniella* is the largest genus in the family Thripidae, with 230 species mostly from Central and South America. *F.occidentalis* has been collected commonly in the Cameron Highlands on many different flower hosts, including roses, orchids, daisy, and chrysanthemum. However, two specimens were collected from *Gerbera* sp. at Penang Butterfly Farm. The species is an important vector of Tomato Spotted Wilt Virus (TSWV) and Impatiens Necrotic Spot Virus (INSV). However, there are very few or perhaps no records of these viruses being transmitted among Malaysian crops.

**Recognition**: Body length about 1.5 mm (female), male is smaller about 1.0 mm in length. Body colour varies from yellow to brown, abdomen sometimes shaded medially in specimens from Malaysia, forewing pale yellow, femora and tibiae yellow; antennal segments III, IV and V basal half pale, segments II and VI–VIII dark brown. Pronotum anterior margin with two pairs of long setae, metanotal campaniform sensilla present. Tergite VIII posterior margin with comb of microtrichia in females but not in males.

### *Frankliniellaschultzei* (Trybom)

This species is widespread in tropical and sub-tropical countries. It is commonly collected in the lowlands of Peninsular Malaysia, breeding on flowers and leaves of many host plants. It has been sampled from flowers of *Bougainvillea* sp., *Jatrophaintegerrima*, and *Plumbagoauriculata*. *Frankliniellaschultzei* is an important vector of Tomato Spotted Wilt Virus (TSWV), although occasionally, it is a useful predator of mites on cotton (Milne and Walter 1997).

**Recognition**: This species apparently exists as two very different colour forms, with the body either brown or yellow. According Gikonyo et al. (2017) from the field surveys in Kenya indicate that the two colour forms of *F.schultzei* could be different species, of which, both showed distinct host preferences, lack of interbreeding and molecular differences. In general, *F.schultzei* is distinguished by the lack of ctenidia on abdominal tergite V, the position of ocellar setae pair III between the posterior ocelli, and the absence from the posterior margin of tergite VIII of a comb of microtrichia.

### *Megalurothripsusitatus* Bagnall

This is the most common and widespread species of the genus in the Oriental area. It breeds in legume flowers and is an important pest on bean crops through feeding in the flowers in south China (Tang et al. 2015).

**Recognition**: Body colour brown; fore tibiae, apices of mid and hind tibiae yellow; antennal segment III slightly yellow, segment IV and V brown; antennal segments III and IV with apical constriction, segment VI unusually elongate; abdominal sternite VII posteromarginal setae S1 situated in front of posterior margin

### *Megalurothipstypicus* Bagnall

There are 13 species currently listed in *Megalurothrips*, but these are difficult to identify due to variation both within and between species. Correct identification of species in this genus requires morphological study and comparison of both sexes. In Peninsular Malaysia, only three species are reported: *M.typicus*, *M.mucunae*, and *M.usitatus*, and the latter species is the one most commonly collected on crops in both highlands and lowlands. Adults of *M.typicus* have been taken from Moraceae, Passifloraceae and Fabaceae, but with no reliable record of the host on which it breeds.

**Recognition**: This species is similar to *M.sjostedti*, the only species in this genus from Africa, but the colour of the forewing and antennae are different (Palmer 1987). *Megalurothripstypicus* is easy to distinguish from the other oriental *Megalurothrips* species, because all three pairs of posteromarginal setae on abdominal sternite VII arise at the posterior margin.

### *Microcephalothripsabdominalis* (Crawford)

This sunflower thrips is widespread worldwide, and is the only species in the genus *Microcephalothrips*. In Peninsular Malaysia, adults of this species have been collected largely from the plant family Asteraceae from *Tagetespatula*, *Cosmoscaudatus*, *Chrysanthemumindicum*, *Tridaxprocumbens*, and *Sphagneticolatrilobata*. Other plant families from which adults have been taken include Heliconiaceae, Orchidaceae, and Acanthaceae.

**Recognition**: Body largely brown except fore tibiae, tarsi, and antennal segment III slightly paler. Based on morphology, it is related to the *Thrips* genus-group, because of the presence of a pair of ctenidia on abdominal segments V–VIII, but no other member of this group has setae on the prosternum (Figure 13). The triangular lobed craspedum on the abdominal tergites is very distinctive character of this species. Pronotum without any long setae, surface smooth without any fine transverse lines. Forewing usually curved and vein setae small.

### *Scirtothripsdorsalis* Hood

This species is widespread from Pakistan to Japan, Taiwan, and northern Australia (Hoddle and Mound 2003), and is now introduced to the Caribbean area and Florida. *S.dorsalis* is known as a vector of Tospovirus, the species often becomes a pest of many different crops including vegetables and cut-flowers in Southeast Asia. It was also found infesting young leaves of ‘Berembang’ tree (*Sonneratiacaseolaris*) in UKM’s green house. This mangrove tree is the host to firefly, *Pteroptyxtener* from Peninsular Malaysia.

**Recognition**: This is amongst the smallest of species in the subfamily Thripinae, with the body of both sexes usually less than 1mm. Body yellow, with all femora, tibiae, and tarsi yellow; antennal segment I pale, segment II variable pale or slightly shaded, segments III–VIII brown; forewings pale yellow to light brown, with apex paler. Abdominal tergites and sternites IV–VII with median brown marking. An identification key to the species of this genus is available in Ng et al. (2014).

### *Stenchaetothripsbiformis* (Bagnall)

This Oriental rice thrips breeds on grasses including paddy, on which crop it is a pest in Philippines and Thailand. However, such infestation has not been reported in Malaysia. This thrips usually lives in moist places like in paddy field and grasses by the pond site.

**Recognition**: Body uniformly brown; antennal segments I–II dark, II paler at apex, III pale, IV slightly shaded, V–VII dark; all tibiae and tarsi pale; fore wing uniformly shaded. Metanotum with closely spaced striations, campaniform sensilla absent, median setae arising behind the anterior margin but varying in position. Meso- and metafurca each without a spinula. Male abdominal sternites III–VII with a transverse pore plate.

### *Thripsflorum* Schmutz

Identification of this species from its close relative, *T.hawaiiensis*, is not easy, and a third related species, *T.razanii*, was described recently from Peninsular Malaysia (Ng et al. 2010). For correct identification of these species it is essential that specimens are well-mounted onto slides. *Thripsflorum* has not been reported as a severe pest in Malaysia’s agriculture sector, but it has been collected from a wide range of flowers.

**Recognition**: Body colour brown; antennae brown except segment III yellow; legs yellow; forewing brown pale at base. Ocellar setae III arising outside ocellar triangle; postocular seta II much shorter than setae I and III. Mesonotum without sculpture lines near campaniform sensilla; metanotum with many lines at the anterior and laterally. Forewing clavus apical seta usually shorter than subapical seta. This species is very similar to both *T.hawaiiensis* and *T.razanii*, and comparisons are given in Ng et al. (2010). More discussion is available in Palmer (1999).

### *Thripshawaiiensis* (Morgan)

This is the most common and abundant species of the genus *Thrips* in Peninsular Malaysia, and is found on a wide range of cultivated plants in various families including Asteraceae, Apocynaceae, and Fabaceae. It is considered the pollinator of oil palm trees, but is reported as a pest of roses in Georgia, citrus in India, coffee and mangoes in Thailand, and banana in Australia (Palmer 1999).

**Recognition**: Body colour brown or bicoloured with head and pronotum paler than brown abdomen. Antennal segment III yellow, segments IV and V usually brown or paler at base; legs yellow; forewing paler at base. More discussion about the variation of this species is available in Palmer (1999) and Ng et al. (2010). A key to the species of *Thrips* genus from Peninsular Malaysia was published by Mound and Azidah (2009).

### *Thripspalmi* Karny

This species is a common pest in Malaysia and also many other Southeast Asian countries. It is a vector of plant tospovisuses, but it can also cause severe feeding damage to the leaves of crops such as eggplant and capsicum (Tan et al. 2016).

**Recognition**: Body colour pale yellow; antennae 7-segmented, segments I and II pale yellow, other segments light brown. The species is easy to distinguish from most *Thrips* species because of the complete posteromarginal comb on abdominal tergite VIII and the lack of discal setae on abdominal sternites. In Malaysia, the species has been taken from crops including *Capsicum*, *Cucumis* and *Solanum* (Mound & Azidah, 2009), as well as species of Rubiaceae.
